# Effects of resistance training on preventing muscle atrophy and bone loss in simulated weightless population: a systematic review and meta-analysis

**DOI:** 10.3389/fphys.2025.1694891

**Published:** 2025-11-14

**Authors:** Aona Chen, Chenggen Guo, Yingcong Zhou

**Affiliations:** 1 School of Physical Education, Wuhan University of Technology, Wuhan, China; 2 Hubei Research Centre for Traditional Ethnic Sports Culture Inheritance and Innovation, Wuhan University of Technology, Wuhan, China; 3 School of Sports Training, Wuhan Sports University, Wuhan, China

**Keywords:** resistance training, microgravity, myasthenia gravis, bone mass, simulatedweightlessness, bed rest

## Abstract

**Objective:**

This systematic review and meta-analysis examined the effects of resistance training on preventing muscle atrophy and bone loss under simulated weightlessness, and identified moderating factors influencing these outcomes.

**Methods:**

PubMed, Web of Science, Scopus, and SPORTDiscus were searched for articles up to October 2024. Study quality was assessed with the PEDro scale, publication bias with funnel plots and Egger’s test, and certainty of evidence with the GRADE approach. A multilevel random-effects meta-analysis and moderator analyses were performed in R.

**Results:**

Eighteen studies (297 participants) were included. Resistance training significantly increased muscle cross-sectional area (CSA) with a large effect (g = 0.95, 95% CI: 0.50–1.39, p < 0.01), with the triceps surae showing the largest CSA gains (g = 2.29). Muscle volume also improved (g = 0.84, 95% CI: 0.57–1.12, p < 0.01), moderated by sex, training type, frequency, and muscle tested. Larger effects were seen in women (g = 2.33), concurrent training (g = 2.33), 2–3 weekly sessions (g = 2.33), and quadriceps (g = 1.62). Muscle strength increased substantially (g = 2.26, 95% CI: 1.42–3.11, p < 0.01), with greater gains in women (g = 3.49), concurrent training (g = 3.08), and 60–70 days of training (g = 2.92). For bone health, resistance training increased bone mineral content (g = 0.73, 95% CI: 0.41–1.05, p < 0.01) and bone formation markers (g = 0.69, 95% CI: 0.31–1.07, p < 0.01), but had no significant effect on bone resorption (g = 0.15, p > 0.01).

**Conclusion:**

Resistance training effectively attenuates muscle atrophy and improves strength, particularly in women, with concurrent training, moderate training frequencies, and 60–70 days programs. Benefits are most evident in the quadriceps and triceps surae. Resistance training also enhances bone mineral content and bone formation, though effects on bone resorption are negligible.

## 1 Introduction

Throughout human evolution, physiological functions have adapted to Earth’s gravitational environment. However, exposure to microgravity or reduced gravity environments (e.g., the Moon’s 1/6 g and Mars’s 1/3 g) leads to locomotor system degradation, which is a major concern for astronaut health and work performance during both short- and long-duration space missions (Wang et al., 2019; Lv et al., 2022). Additionally, individuals on Earth, such as those who are bedridden or experience limb immobilization, are also susceptible to similar physiological decline. Addressing the challenge of weightlessness-induced motor system degradation is therefore an urgent and necessary priority.

The musculoskeletal system provides the structural foundation for human morphology, stability, and movement. Research indicates that musculoskeletal degradation in microgravity is a major limitation for astronauts’ on-orbit performance, primarily manifesting as muscle atrophy and bone loss (Lee et al., 2022; Stein, 2013). In microgravity, a decrease in muscle protein synthesis and an increase in protein catabolism contribute to reduced muscle volume, decreased cross-sectional area, loss of muscle mass, and declining strength. Flight studies have shown that exposure to microgravity for 7–15 days leads to moderate muscle atrophy, while long-term spaceflight (6 months) results in an 8.8%–15.9% reduction in plantarflexor muscle volume, a 35%–40% decline in neuromuscular activity, and a 17% decrease in maximal isometric muscle strength (Winnard et al., 2019; Guo et al., 2018). Bone tissue plays a crucial role in weight support, organ protection, and movement, and its metabolism is regulated by the coordinated activity of osteoblasts and osteoclasts. In microgravity, the absence of daily gravitational loads increases astronauts’ susceptibility to bone loss during space missions. Studies show that bone loss severity escalates with longer flight durations, occurring at a rate faster than that observed in menopausal women (Juhl et al., 2021). Another study reported that astronauts in weightlessness lose 1.5%–2% of their bone mass per month (Thornton and Bonato, 2017). Muscle atrophy and bone loss in microgravity may impair astronauts’ ability to perform missions and increase the risk of injury upon returning to Earth’s gravity. Therefore, developing effective protective strategies and countermeasures against weightlessness-induced musculoskeletal degradation is essential.

Resistance training is the primary countermeasure against the effects of microgravity on astronauts. However, due to the limited research opportunities in space, constraints such as small spacecraft payloads and the low number of astronauts, most findings on resistance training stem from ground-based simulated weightlessness experiments rather than real-space conditions. Despite their value, ground-based studies face several limitations that hinder a comprehensive understanding of resistance training’s efficacy in preventing muscle atrophy and bone loss and reduce their applicability to practical countermeasures. First, inconsistencies in study design, variable study quality, and small sample sizes (Mulder et al., 2009) have led to divergent findings. For instance, Akima et al. (Akima et al., 2001) assigned participants to a control group (n = 10) and an experimental group (n = 5). After 20 days of resistance training during 6° head-down tilt bed rest, no significant differences in calf muscle cross-sectional area were observed between groups, questioning the intervention’s effectiveness. Second, different weightlessness simulation paradigms, such as dry immersion and bed rest, contribute to variability in experimental outcomes (Song et al., 2023). Dry immersion uses water buoyancy and hydrostatic pressure to simulate the physiological effects of weightlessness, with subjects sitting or lying in a water tank containing 1%–2% salt content. In contrast, bed rest protocols simulate weightlessness through prolonged immobilization, with head-down tilt angles ranging from 0° to–12°. Variations in these methods introduce inconsistencies in study results. Third, there is a lack of research examining the moderating effects of key variables, such as sex, resistance training type, intervention duration, and training frequency, on muscle atrophy and bone mass loss. This gap limits the ability to develop evidence-based guidelines for effective countermeasures. Addressing these challenges through standardized study designs, larger sample sizes, and controlled simulation methods is crucial for enhancing the efficacy of resistance training in spaceflight conditions.

Given the limitations in existing research, we conducted a systematic literature review. However, few systematic reviews or meta-analyses have specifically examined the effects of resistance training on muscle atrophy and bone loss in simulated weightless populations. This gap underscores the novelty and significance of our study. Notably, our findings may also provide valuable insights for rehabilitation strategies in populations experiencing similar conditions on Earth, such as long-term bedridden patients or individuals with immobilized limbs, thereby broadening the application of resistance training in sports medicine. Bed rest serves as an established model for simulating weightlessness, as it induces body mass reduction, tissue fluid redistribution, altered hydrostatic pressure, and reduced sensory input—physiological responses closely resembling those observed in microgravity environments (Hargens and Richardson, 2009). Therefore, we adopted bed rest as the primary paradigm for our study. Additionally, research comparing head-down tilt (HDT) angles (0°, −4°, −6°, −8°, and −12°) has identified −6° HDT-bed rest (HDT-BR) as the most effective model for replicating the fluid shifts and physiological adaptations experienced in space (Traon et al., 2007; Hargens, 1994). Based on these findings, our study employed −6° HDT-BR to simulate weightlessness and assess the effects of resistance training on mitigating muscle atrophy and bone loss. Furthermore, we investigated the moderating effects of key variables, including sex, resistance training type, intervention duration, training frequency, and targeted muscle groups, to refine evidence-based recommendations for counteracting musculoskeletal degradation in microgravity-like conditions.

## 2 Methods

This study protocol was registered with PROSPERO (Preferred Reporting Items for Systematic Review and Meta-Analysis Protocols) on 17 July 2024 (CRD42024569115). The review was conducted and reported in accordance with PRISMA (Preferred Reporting Items for Systematic Reviews and Meta-Analyses) and PERSiST (Implementing PRISMA in Exercise, Rehabilitation, Sport Medicine, and Sports Science) guidelines (Ardern et al., 2022; Page et al., 2021).

### 2.1 Search strategy

A comprehensive literature search was conducted in PubMed, Web of Science (all databases), Scopus, and SPORTDiscus. All articles indexed up to July 2024 were considered for inclusion. Various search terms and Boolean operators were applied [see Supplementary Table S1 in the Online Supplementary Material (OSM)]. No language restrictions were imposed during the search. The search was updated in October 2024 through database alerts identifying newly indexed studies. Additionally, reference lists from eligible studies, systematic reviews, and meta-analyses retrieved from our search were examined for relevant articles. Two independent reviewers (AC,CG) screened the titles, abstracts, and full texts of all identified articles. Discrepancies were resolved through consultation with a third independent reviewer (YZ). Ultimately, 18 studies were included in the systematic review and meta-analysis.

### 2.2 Eligibility criteria

Only peer-reviewed studies published in scientific journals were included, without any language restrictions. Gray literature, conference papers, and dissertations were excluded. In accordance with the PRISMA guidelines, the PICOS framework (Population, Intervention, Comparators, Outcomes, and Study Design) was applied to assess study eligibility (Amir-Behghadami and Janati, 2020).

#### 2.2.1 Population

A healthy simulated weightless population was included without restrictions on race, nationality, age, or gender. Participants underwent strict bed rest with a −6° head-down tilt (HDT-BR) to replicate the microgravity environment of space (Traon et al., 2007; Hargens, 1994). Individuals with musculoskeletal disorders, including sarcopenia, myositis, osteoporosis, or osteoarthritis, were excluded. Additionally, studies utilizing dry immersion or alternative ground-based methods to simulate weightlessness, rather than −6° HDT-BR, were not considered.

#### 2.2.2 Intervention

The experimental group performed resistance training—including traditional, flywheel, or concurrent training—with various movements (e.g., squats, deadlifts, stirrups, heel raises) and all contraction types (concentric, eccentric, isometric). Based on prior research, at least 3–4 weeks are typically needed to induce muscle and bone adaptations in weightlessness (Lambertz et al., 2001; Smith et al., 2012); thus, only studies with interventions ≥20 days were included.

#### 2.2.3 Comparators

The control group consisted of participants undergoing bed rest, nutritional supplementation, medication, or placebo interventions. Studies in which the control group engaged in any form of exercise, such as balance training, core training, or stretching, were excluded.

#### 2.2.4 Outcomes

Outcomes were categorized into primary and secondary indicators. The primary outcome indicator was muscle atrophy, assessed through muscle cross-sectional area, muscle volume, and muscle strength. The secondary outcome indicator was bone quality, evaluated using bone mineral content, bone formation markers, and bone resorption markers. Bone formation markers included alkaline phosphatase (ALP), bone-specific alkaline phosphatase (b-ALP), osteocalcin (OC), and procollagen type I N-terminal propeptide (P1NP). Bone resorption markers included C-terminal cross-linked telopeptide (CTX), N-terminal cross-linked telopeptide of type I collagen (NTX), tartrate-resistant acid phosphatase (TRAP), pyridinoline (PYD), deoxypyridinoline (DPD), and others.

#### 2.2.5 Study design

Only randomized and non-randomized controlled studies were included; cross-sectional, observational, and case studies were excluded.

### 2.3 Data extraction

Data were extracted by one author (AC) using a Microsoft Excel template and verified by a second author (CG), including study details (first author, year), participant characteristics (age, sex, sample size), training variables (type, duration, frequency), and outcomes. Discrepancies were resolved by a third author (YZ). Baseline and post-intervention means and SDs were extracted to calculate effect sizes; when unavailable or improperly reported, corresponding authors were contacted, and studies with missing data were excluded. Study characteristics are summarized in Table 1.

**TABLE 1:** Characteristics of the included studies.

Study	Population	Intervention	Comparison	Outcomes	Study design
Rittweger and Felsenberg, (2009) United Kingdom	18 healthy men (32.0 ± 4.2 years; 174.6 ± 4.1cm; 71.1 ± 6.2 kg) 90 days of bed rest with −6° head down tilt (HDT-BR)	Flywheel Resistive Exercise (FW) Participants performed FW training 2–3 times per week, including supine squats (4 × 7 reps) and calf raises (4 × 14 reps), with a progressive warm-up and structured rest intervals	bed-rest only	mCSA BMC ALP	RCT
Rittweger et al. (2005) Germany	18 healthy men (32.0 ± 4.2 years; 175.0 ± 4.1cm; 71.1 ± 6.2 kg) 90 days of bed rest with −6° head down tilt (HDT-BR)	Flywheel Resistive Exercise (FW) Participants performed FW training 2–3 times per week, starting with a progressive warm-up, then completing squats (4 × 7 maximal concentric–eccentric reps) and calf presses (4 × 14 reps), with 2-min rests between sets and 5-min rests between exercises	bed-rest only	mCSA BMC ALP PYD	RCT
Holt et al. (2016) USA	16 healthy women (33.0 ± 1 years; 164.9 ± 2.5cm; 58.1 ± 2.2 kg) 60 days of bed rest with −6° head down tilt (HDT-BR)	Concurrent Training (CT) Protocol The CT protocol consisted of aerobic exercise (40%–80% VO_2_peak) and flywheel resistive training, including leg press (4 × 7 maximal concentric–eccentric reps) and calf press (4 × 14 reps), with 2-min rests between sets, performed 2–3 times per week	bed-rest only	mCSA MS	RCT
Akima et al. (2000) Japan	9 healthy men (24.0 ± 4.7years; 173.0 ± 4.6cm; 69.9 ± 11 kg) 20 days of bed rest with −6° head down tilt (HDT-BR)	Resistance Training (RT) Participants performed daily isometric leg presses (7 sessions/week), each with 30 repetitions of 3-s contractions and 3-s rests, maintaining joint angles of \∼80° (ankle), 90° (knee), and 110° (hip)	bed-rest only	mCSA MV	RCT
Akima et al. (2001) Japan	15 healthy men (23.8 ± 4.2years; 173.5 ± 4.9cm; 68.5 ± 10.3 kg) 20 days of bed rest with −6° head down tilt (HDT-BR)	Resistance Training (RT) Participants performed resistance training (RT) twice daily, 7 days per week: a morning leg press session (3 × 10 reps, 1-min rest) and an afternoon isotonic leg press at 40% max load to exhaustion, with joint angles of \∼110° (hip), 90° (knee), and 80° (ankle)	bed-rest only	mCSA	No-RCT
Akima et al. (2003) Japan	12 healthy men (23.3 ± 2.0years; 169.8 ± 2.6cm; 65.5 ± 7.0 kg) 20 days of bed rest with −6° head down tilt (HDT-BR)	Resistance Training (RT) Participants completed resistance training 7 days per week with morning leg press and afternoon bilateral plantar flexion sessions, each consisting of five sets of 10 reps at 70% maximal isometric force with 1-min rests	bed-rest only	mCSA	No-RCT
Mulder et al. (2009) Netherlands	16 healthy men (31.1 ± 5.1years; 179.3 ± 7.7cm; 75.0 ± 12.8 kg) 60 days of bed rest with −6° head down tilt (HDT-BR)	Resistance Training (RT) Participants performed resistance training three times per week, including dynamic bilateral squats, unilateral and bilateral calf raises, and bilateral static back extensions	bed-rest only	mCSA MS	RCT
Ploutz-Snyder et al. (2018) USA	17 healthy men and women (33.0 ± 10.0years; 77.0 ± 14.0 kg) 70 days of bed rest with −6° head down tilt (HDT-BR)	Concurrent Training (CT) Concurrent training (CT) involved alternating-day aerobic exercise—either 30 min of continuous cycling at 75% VO_2_ peak or near-maximal interval treadmill sessions of 30 s, 2 min, or 4 min—combined with resistance training consisting of three sets each of supine squat, leg press, unilateral leg curl, and heel raise	sedentary	mCSA MS b-ALP OC DPD NTX	RCT
Trappe et al. (2007) USA	16 healthy women (33.0 ± 1.0years; 165.0 ± 3.0cm; 58.1 ± 2.2 kg) 60 days of bed rest with −6° head down tilt (HDT-BR)	Concurrent Training (CT) CT was performed three times per week, combining flywheel resistance training—supine squat and calf press, four sets of 14 maximal concentric-eccentric reps with 2-min rests—and supine treadmill aerobic exercise at 40%–80% VO_2_ peak	bed-rest only	MV MS	No-RCT
Alkner and Tesch, (2004a) Sweden	17 healthy men (33.0 ± 5.0years; 176.0 ± 5.0cm; 71.0 ± 6.0 kg) 29 days of bed rest with −6° head down tilt (HDT-BR)	Flywheel Resistive Exercise (FW) FW training was performed three times per week using two flywheels (44 cm, 2.5 kg each, total inertia 0.1105 kg m^2^) for supine squats (four sets of 7 reps) and calf presses (four sets of 14 reps), with 2-min rests between sets and 5 min between exercises	bed-rest only	MV	No-RCT
Alkner and Tesch, (2004b) Sweden	17 healthy men (33.0 ± 5.0years; 176.0 ± 5.0cm; 71.0 ± 6.0 kg) 90 days of bed rest with −6° head down tilt (HDT-BR)	Flywheel Resistive Exercise (FW) FW training was performed three times per week, including supine squats (4 × 7 reps) and calf presses (4 × 14 reps) with 2-min rests between sets and 5 min between exercises	bed-rest only	MV MS	No-RCT
Belavý et al. (2017) Germany	25 healthy men (31.0 ± 5.5years; 175.0 ± 5.0cm; 70.9 ± 5.4 kg) 90 days of bed rest with −6° head down tilt (HDT-BR)	Flywheel Resistive Exercise (FW) The flywheel group trained 2–3 times per week, performing supine leg press (4 × 7 reps, 5-min rest) and calf raises (4 × 14 reps, 2-min rests between sets)	bed-rest only	MV	RCT
Rittweger et al. (2013) Germany	24 healthy men (32.5 ± 3.4 years; 174.2 ± 3.9cm; 71.4 ± 6.7 kg) 90 days of bed rest with −6° head down tilt (HDT-BR)	Flywheel Resistive Exercise (FW) Training was performed 2–3 times per week, including supine squats (4 × 7 maximal reps) and calf presses (4 × 14 maximal reps) with 2-min rests between sets and 5 min between exercises	bed-rest only	mCSA	RCT
Kouzaki et al. (2007) Japan	12 healthy men (23.3 ± 4.9years; 169.8 ± 6.4cm; 65.5 ± 17.1 kg) 20 days of bed rest with −6° head down tilt (HDT-BR)	Resistance Training (RT) Training was performed seven times per week with morning leg press and afternoon calf raise sessions, each consisting of five sets of 10 dynamic bilateral repetitions (1 s shortening, 2 s lengthening) with 60-s rests	bed-rest only	MV	RCT
Miokovic et al. (2011) Germany	17 healthy men (32.5 ± 3.4years; 174.0 ± 4.0cm; 70.3 ± 6.1 kg) 60 days of bed rest with −6° head down tilt (HDT-BR)	Resistance Training (RT) RT was performed three times per week, including bilateral squats at 75%–80% of pre-bedrest MVC and single-leg heel raises with 1.3× body weight	bed-rest only	MV	RCT
Miokovic et al. (2014) Germany	16 healthy men (31.1 ± 5.1years; 179.3 ± 7.7cm; 75.0 ± 12.8 kg) 60 days of bed rest with −6° head down tilt (HDT-BR)	Resistance Training (RT) RT was performed three times per week, including bilateral leg press (75%–80% pre-bedrest MVC), single-leg (1.3× body weight) and double-leg (1.8× body weight) heel raises, and back/forefoot raises (1.5× body weight), with each session lasting 5–6 min and a total weekly time of 22 min including rests	bed-rest only	MV	RCT
Lee et al. (2014) USA	16 healthy women (33.0 ± 1.0years; 164.9 ± 2.5 cm; 58.1 ± 2.2 kg) 60 days of bed rest with −6° head down tilt (HDT-BR)	Concurrent Training (CT) Subjects performed CT three times per week, combining aerobic exercise at 40%–80% of pre-bedrest VO_2_peak with flywheel leg press (4 × 7 maximal reps) and calf press (4 × 14 maximal reps)	bed-rest only	MS	No-RCT
Smith et al. (2008) USA	16 healthy women (32.0 ± 4.0years; 166.0 ± 7.0 cm; 59.0 ± 5.0 kg) 60 days of bed rest with −6° head down tilt (HDT-BR)	Flywheel Resistive Exercise (FW) Subjects performed FW training 2–3 times per week, including supine leg press (4 × 7) and calf press (4 × 14) maximal concentric–eccentric repetitions, with 2-min rests between sets	bed-rest only	BMC ALP b-ALP OC PINP NTX CTX DPD TRAP	RCT

RCT, randomized controlled trial; mCSA, muscle cross sectional area; BMC, bone mineral content; ALP, alkaline phosphatase; PYD, pyridine; MS, muscle strength; MV, muscle volume, b-ALP, bone specific alkaline phosphatase; OC, osteocalcin; DPD, deoxy pyridinoline; NTX, amino-terminal cross-linked telopeptide of type 1 collagen, P1NP, procollagen type Ⅰ N-prepeptide,CTX c-tenninal cross linked peptide, TRAP, tartrate resistant acid phosphatase.

### 2.4 Methodological quality

The quality of the included studies was assessed using the Physiotherapy Evidence Database (PEDro) scale (De Morton, 2009; Maher et al., 2003). The PEDro scale comprises 11 items, which assess eligibility criteria, random allocation, allocation concealment, comparability of baseline groups, blinding of patients, therapists, and assessors, analysis by intention-to-treat, between-group statistical comparisons, and point measures with variability data. Notably, for this systematic review, items five to seven of the PEDro scale were excluded, as blinding of subjects, assessors, and researchers is infrequent in supervised exercise interventions (Maher et al., 2003; González-Mohíno et al., 2020).

Each criterion on the PEDro scale was rated as “1” (indicating that the criterion was met) or “0” (indicating that the criterion was not met). Based on previous studies (Maher et al., 2003), the studies were categorized as follows: ≥6 points = “high quality,” 4–5 points = “moderate quality,” and ≤3 points = “low quality.” The quality of each study was independently assessed by two reviewers (CG and YZ), with an intraclass inter-rater correlation coefficient of 94.4%. In case of disagreement, a third reviewer (AC or YZ) was consulted. The total PEDro scores for the included studies are presented in Table 2.

**TABLE 2:** Methodological quality assessment.

Authors, year	N1	N2	N3	N4	N8	N9	N10	N11	Total	Quality assessment
Rittweger and Felsenberg. (2009)	1	1	0	1	1	0	1	1	6	high quality
Rittweger et al. (2005)	1	1	0	1	1	1	1	1	7	high quality
Holt et al. (2016)	1	1	0	1	1	1	0	1	6	high quality
Akima et al. (2000)	1	1	0	1	1	1	1	1	7	high quality
Akima et al. (2001)	1	0	0	1	0	1	1	1	5	moderate quality
Akima et al. (2003)	1	0	0	1	0	1	1	1	5	moderate quality
Mulder et al. (2009)	1	1	0	1	1	1	1	1	7	high quality
Ploutz-Snyder et al. (2018)	1	1	0	1	1	1	1	1	7	high quality
Trappe et al. (2007)	1	0	0	1	1	1	1	0	6	high quality
Alkner and Tesch, (2004a)	1	0	0	1	0	1	1	1	5	moderate quality
Alkner and Tesch, (2004b)	1	0	0	1	0	1	1	1	5	moderate quality
Belavý et al. (2017)	1	1	0	1	1	0	1	1	6	high quality
Rittweger et al. (2013)	1	1	0	1	1	1	1	1	7	high quality
Kouzaki et al. (2007)	1	1	0	1	1	1	1	1	7	high quality
Miokovic et al. (2011)	1	1	0	1	1	1	1	1	7	high quality
Miokovic et al. (2014)	1	1	0	1	1	1	1	1	7	high quality
Lee et al. (2014)	1	0	0	1	1	0	1	0	4	moderate quality
Smith et al. (2008)	1	1	0	1	0	1	0	1	5	moderate quality

N1: eligibility criteria were specified; N2: subjects were randomly allocated to groups; N3: allocation was concealed; N4: the groups were similar at baseline regarding the most important prognostic indicators; N8: measures of at least one key outcome were obtained from more than 85% of the subjects initially allocated to groups; N9: all subjects for whom outcome measures were available received the treatment or control condition as allocated or, where this was not the case, data for at least one key outcome was analysed by “intention to treat”; N10: the results of between-group statistical comparisons are reported for at least one key outcome; N11: the study provides both point measures and measures of variability for at least one key outcome; Risk of bias: ≥6 points = “high quality”, 4 to 5 points = “moderate quality”, and ≤3 points = “low quality”. Items 5 to 7 of the original scale were removed due to the infrequency of blinding of subjects, evaluators, and researchers in supervised exercise interventions.

### 2.5 Statistical analysis

All analyses were conducted using the statistical software R with the “metaSEM” and “metafor” packages. Since the outcome indicators involved multiple test units, standardized mean difference (SMD) was prioritized as the effect size indicator, based on prior research recommendations (Takeshima et al., 2014). Additionally, given the small sample size in most of the included studies, Hedges’ g, calculated using the exact formula, was used as the effect size indicator.

A positive Hedges’ g indicates an increase in muscle and bone in the experimental group relative to the control group. According to conventional guidelines, a Hedges’ g value of 0.2 is considered a small effect size, 0.5 is considered moderate, and 0.8 is considered large (Hedges and Olkin, 1985). Outcomes across studies were pooled using a random effects model. Additionally, a prediction interval was calculated to assess the potential variability of resistance training effects when applied in individual study settings, as these may differ from the average effect (Riley et al., 2011).

Between-study heterogeneity was assessed using τ^2^ (the variance of true effects) and the I^2^ statistic, which quantifies the proportion of between-study variance relative to the total observed variance (Higgins et al., 2003). An I^2^ value of 75% was considered large, 50% moderate, and 25% low. If moderate to high heterogeneity was detected, a moderator variable test was conducted on the outcome indicator. Additionally, the Egger’s regression intercept test and visual inspection of the funnel plot were employed to identify potential publication bias (Egger et al., 1997). If publication bias was present (p < 0.1 on the Egger’s test), further adjustments were made using the trim and fill method (Duval and Tweedie, 2000). In this study, a statistically significant difference was defined as a p-value less than 0.05, while a p-value between 0.05 and 0.10 was considered indicative of a statistically significant trend.

### 2.6 Grading the quality of evidence

Although meta-analysis is a powerful tool for synthesizing evidence, not all overall effect sizes are meaningful. Therefore, evaluating the strength and quality of the evidence is essential. The GRADE (Grading of Recommendations, Assessment, Development, and Evaluation) system is widely recognized for assessing the quality of evidence and the strength of recommendations (Alonso-Coello et al., 2016). The GRADE approach was applied to rate the certainty of the evidence in this systematic review (Guyatt et al., 2011).

The GRADE system classifies evidence quality as high, moderate, low, or very low, reflecting the likelihood that further research could change treatment effect estimates. RCTs start as high-quality evidence, while non-RCTs start as low, with both subject to adjustment based on five downgrading factors (inconsistency, indirectness, imprecision, publication bias, other considerations) and three upgrading factors (large effect size, control for confounders, dose-response relationship). This system was used to assess the reliability of the outcome measures in this study.

## 3 Results

### 3.1 Study selection

**FIGURE 1:**
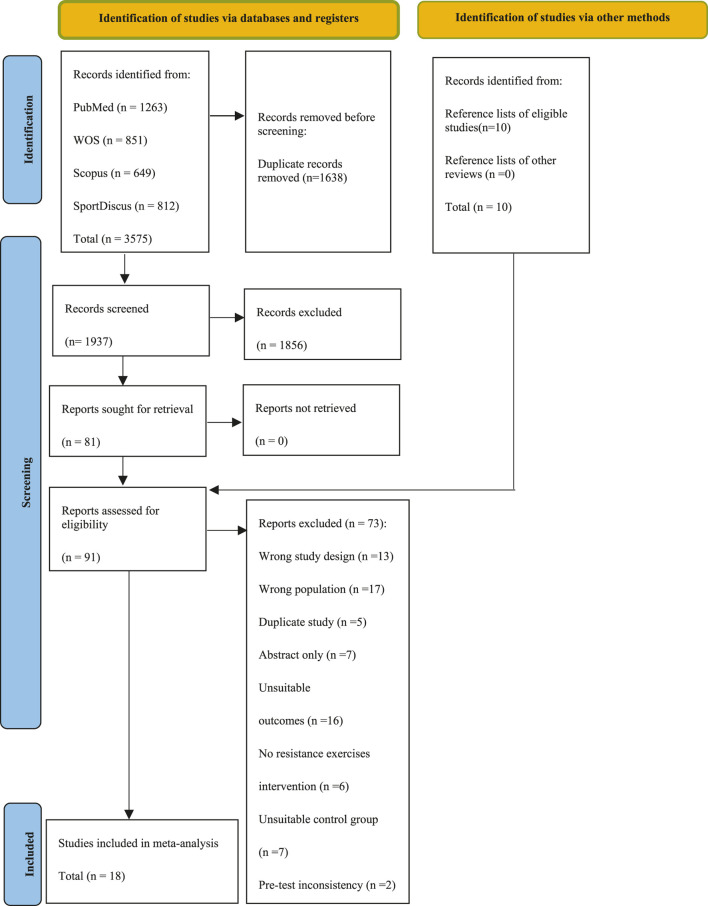
Preferred Reporting Items for Systematic Reviews and Meta-Analyses (PRISMA) fow diagram for the identifcation, screening and inclusion of studies.

Figure 1 shows the literature search and screening process, including reasons for full-text exclusions. A total of 3,575 records were identified (PubMed: 3,416; Web of Science: 851; Scopus: 649; SPORTDiscus: 812). After removing duplicates, inaccessible records, and those excluded during title/abstract screening, 91 studies were assessed for eligibility. Following full-text screening, 18 studies were ultimately included in the meta-analysis (Mulder et al., 2009; Akima et al., 2000; 2001; 2003; Rittweger et al., 2005; Rittweger and Felsenberg, 2009; Rittweger et al., 2013; Holt et al., 2016; Ploutz-Snyder et al., 2018; Trappe et al., 2007; Alkner and Tesch, 2004a; 2004b; Belavý et al., 2017; Kouzaki et al., 2007; Miokovic et al., 2011; 2014; Lee et al., 2014; Smith et al., 2008) (Figure 1; Table 1).

### 3.2 Study characteristics

Of the 18 studies included, the majority were published in the USA (n = 5) (Holt et al., 2016; Ploutz-Snyder et al., 2018; Trappe et al., 2007; Lee et al., 2014; Smith et al., 2008) and Germany (n = 5) (Rittweger et al., 2005; Belavý et al., 2017; Rittweger et al., 2013; Miokovic et al., 2011; Miokovic et al., 2014), followed by Japan (n = 4) (Akima et al., 2001; Akima et al., 2000; Akima et al., 2003; Kouzaki et al., 2007), Sweden (n = 2) (Alkner and Tesch, 2004a; Alkner and Tesch, 2004b), the Netherlands (n = 1) (Mulder et al., 2009), and the United Kingdom (n = 1) (Rittweger and Felsenberg, 2009). Of the included studies, 13 involved male participants, four involved female participants, and one included both sexes. All participants underwent −6° head-down tilt bed rest (HDT-BR) to simulate weightlessness. Interventions comprised Flywheel Resistance Exercise (FW; seven studies), Resistance Training (RT; seven studies), and Concurrent Aerobic and Resistance Training (CT; four studies), lasting 20–90 days with 2–7 sessions per week. Control groups mainly underwent bed rest only.

### 3.3 Methodological quality assessment

As shown in Table 2, 12 of the included studies were of high quality, and six were of moderate quality. The experimental design employed randomized grouping in 12 articles, while six articles did not utilize randomized grouping. All studies included specific participant eligibility criteria, but none of the studies performed allocation concealment. Baseline data were consistent between the experimental and control groups in all studies. The PEDro score analysis revealed scores ranging from four to 7, with a mean score of 6.06, indicating that the overall quality of the included literature was high.

### 3.4 Main efects

#### 3.4.1 Meta-analysis of the preventive effect of muscle atrophy

**FIGURE 2:**
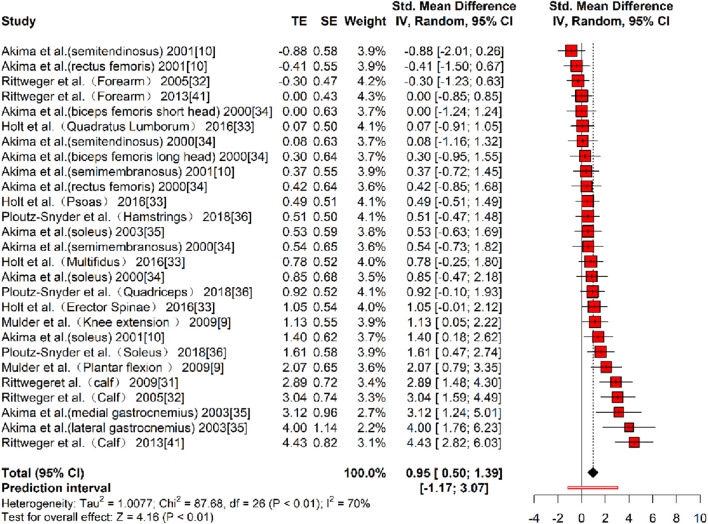
Forest plot of mCSA results (exercise *versus* control group) after resistance training (pre *versus* post). The values in parentheses indicate the analyzed muscle in each study.

**FIGURE 3:**
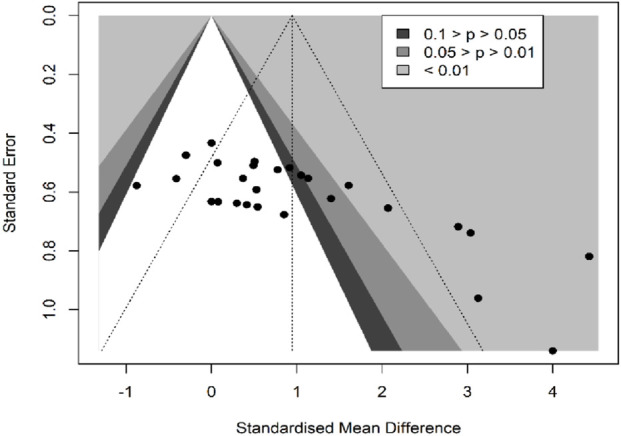
Funnel plot of mCSA results (exercise *versus* control group) after resistance training (pre *versus* post).

**FIGURE 4:**
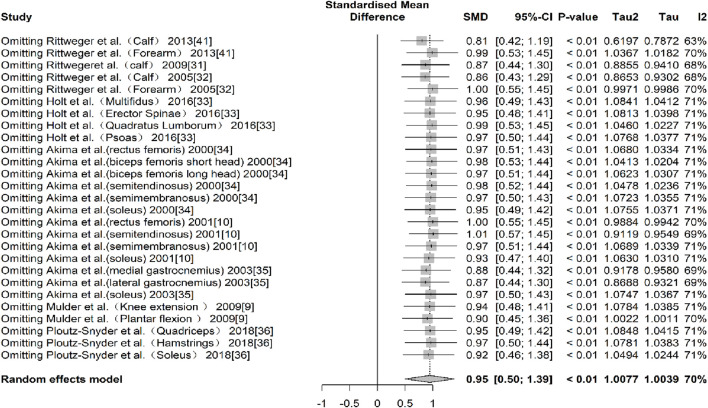
Sensitivity analyses of mCSA results (exercise *versus* control group) after resistance training (pre *versus* post). The values in parentheses indicate the analyzed muscle in each study.

In terms of muscle cross-sectional area (mCSA), nine studies (Mulder et al., 2009; Akima et al., 2001; Rittweger and Felsenberg, 2009; Rittweger et al., 2005; Holt et al., 2016; Akima et al., 2000; Akima et al., 2003; Ploutz-Snyder et al., 2018; Rittweger et al., 2013), reporting 27 effect sizes, were included in the quantitative synthesis, with a total of 405 participants. The overall Hedges’ g indicated a large effect size (k = 27, g = 0.95, 95% CI 0.50–1.39, p < 0.01) with moderate heterogeneity (τ^2^ = 1.008, p < 0.01, I^2^ = 70%). The prediction interval ranged from g = −1.17 to 3.07, suggesting that the effect size could vary substantially across different settings (Figure 2). The funnel plot was used to test for publication bias, and the plot showed significant asymmetry (Figure 3). Further quantitative analysis with Egger’s test confirmed the presence of publication bias (t = 5.01, p < 0.01). A correction was made using the Trim and Fill Method (TFM) to estimate the true effect size. After correction, the meta-analysis was re-run, yielding a combined medium effect size (k = 32, g = 0.73, 95% CI 0.24–1.22, p < 0.01), which was lower than the original estimate, suggesting that the effect of resistance training on muscle cross-sectional area may have been overestimated. Sensitivity analyses indicated that excluding the two studies with the largest (Rittweger et al., 2013) and smallest (Akima et al., 2001) effect sizes, or excluding each study individually (Figure 4), and recombining the effect sizes did not significantly alter the overall results, suggesting that the findings of this study are stable and reliable.

**FIGURE 5:**
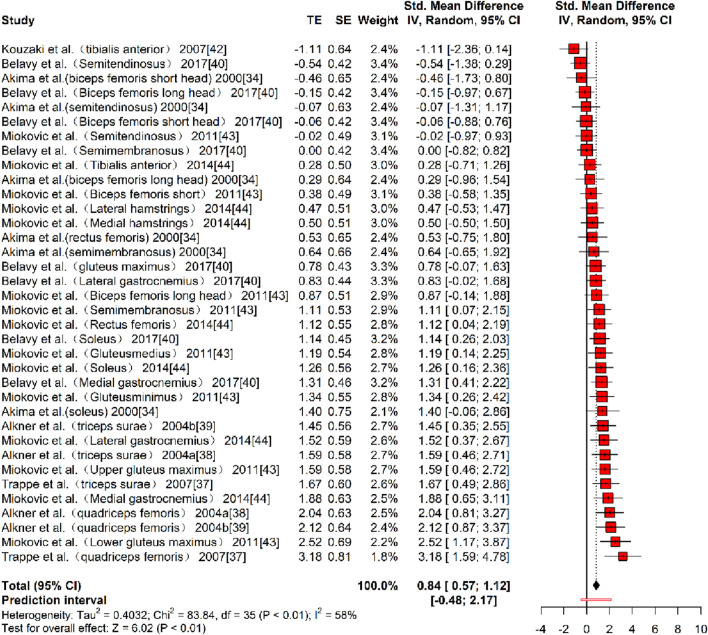
Forest plot of MV results (exercise *versus* control group) after resistance training (pre *versus* post). The values in parentheses indicate the analyzed muscle in each study.

**FIGURE 6:**
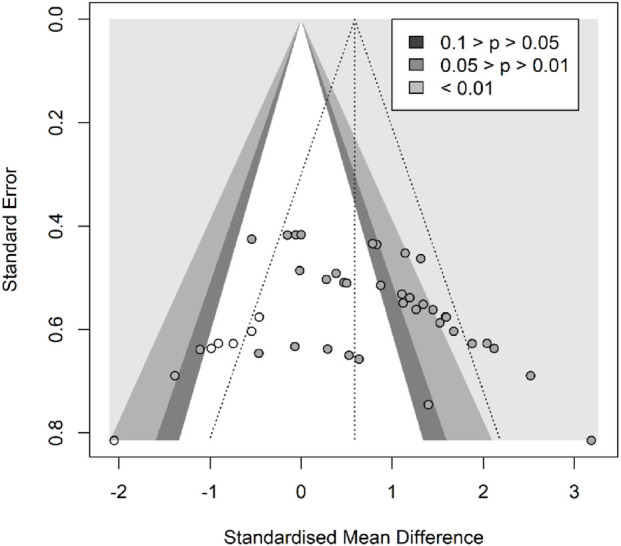
Corrected funnel plot of MV results (exercise *versus* control group) after resistance training (pre *versus* post).

In terms of muscle volume (MV), eight studies (Akima et al., 2000; Trappe et al., 2007; Alkner and Tesch, 2004a; Alkner and Tesch, 2004b; Belavý et al., 2017; Kouzaki et al., 2007; Miokovic et al., 2011; Miokovic et al., 2014), reporting 36 effect sizes, were included in the quantitative synthesis, with a total of 620 participants. The overall Hedges’ g indicated a large effect size (k = 36, g = 0.84, 95% CI 0.57–1.12, p < 0.01) with moderate heterogeneity (τ^2^ = 0.403, p < 0.01, I^2^ = 58%). The prediction interval ranged from g = −0.48 to 2.17, suggesting that the effect size could vary substantially across different settings (Figure 5). The funnel plot bias test showed a largely symmetrical plot. However, Egger’s test revealed significant publication bias (t = 3.36, p < 0.01). To address this, a correction was made using the Trim and Fill Method (TFM) to estimate the true effect size. After correction, the combined results still showed a large effect size (k = 43, g = 0.89, 95% CI 0.61–1.19, p < 0.01). The corrected funnel plot, presented in Figure 6, suggests that publication bias had minimal impact on the results, and that the intervention’s effect on muscle volume is close to the true effect. The sensitivity analysis demonstrated that excluding each study individually resulted in the minimum pooled effect size of g = 0.80 (95% CI 0.53–1.07, k = 35, p < 0.01) when the study by Miokovic et al. (2011) on the lower gluteus maximus was excluded, and the maximum pooled effect size of g = 0.89 (95% CI 0.62–1.16, k = 22, p < 0.01) when the study by Belavý et al. (2017) on the semitendinosus was excluded. Both values were within a reasonable range, suggesting that the results of this study are stable and reliable.

**FIGURE 7:**
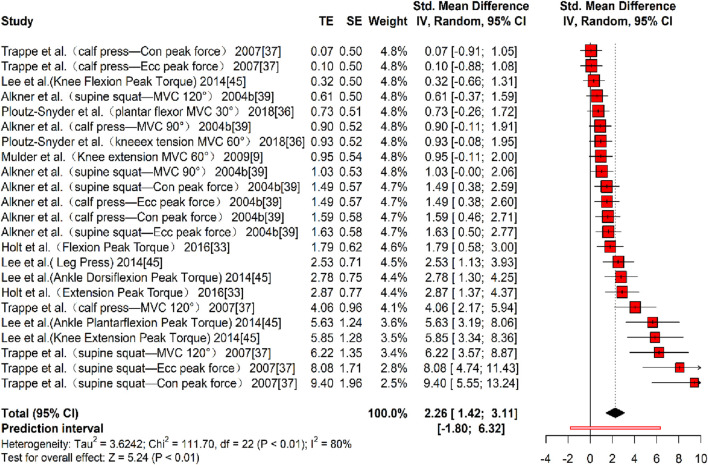
Forest plot of MS results (exercise *versus* control group) after resistance training (pre *versus* post). The values in parentheses indicate the primary muscle group involved in each study.

In terms of muscle strength, six studies (Mulder et al., 2009; Holt et al., 2016; Ploutz-Snyder et al., 2018; Trappe et al., 2007; Alkner and Tesch, 2004b; Lee et al., 2014) reporting 23 effect sizes were included in the quantitative synthesis, involving a total of 377 participants. The overall Hedges’ g indicated a large effect size (k = 23, g = 2.26, 95% CI 1.42–3.11, p < 0.01), with substantial heterogeneity (τ^2^ = 3.624, p < 0.01, I^2^ = 80%). The prediction interval ranged from g = −1.80 to 6.32, suggesting that the effect size may vary considerably across settings (Figure 7). The funnel plot indicated asymmetry, and Egger’s test confirmed publication bias (t = 17.54, p < 0.01). A correction was applied using the Trim and Fill Method (TFM) to account for missing studies, resulting in a corrected large effect size (k = 25, g = 2.31, 95% CI 1.99–2.62, p < 0.01), indicating that publication bias did not significantly affect the results. Sensitivity analysis showed that excluding each study individually resulted in a minimum pooled effect size of g = 2.08 (95% CI 1.29–2.87, k = 22, p < 0.01) when the study by Trappe et al. (2007) (supine squat - MVC 120°) was excluded, and a maximum pooled effect size of g = 2.37 (95% CI 1.49–3.24, k = 22, p < 0.01) when the study by Lee et al. (2014) (Knee Flexion Peak Torque) was excluded. Both values fell within a reasonable range, supporting the stability and reliability of the results.

#### 3.4.2 Meta-analysis of the preventive effect of bone reduction

**FIGURE 8:**
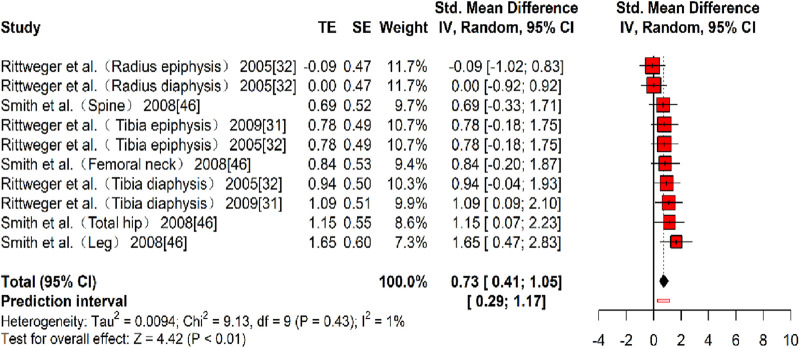
Forest plot of BMC results (exercise *versus* control group) after resistance training (pre *versus* post). The values in parentheses indicate the primary bone site analyzed in each study.

Three studies (Rittweger and Felsenberg, 2009; Rittweger et al., 2005; Smith et al., 2008), reporting 10 effect sizes, evaluated the effect of resistance training on bone mineral content (BMC), with a total of 172 participants. The overall Hedges’ g indicated a large effect size (k = 10, g = 0.73, 95% CI 0.41–1.05, p < 0.01) with negligible heterogeneity (τ^2^ = 0.009, p > 0.01, I^2^ = 1%). The prediction interval ranged from g = 0.29 to 1.17, indicating that the effect size may vary considerably across different settings (Figure 8). The funnel plot suggested no significant publication bias, as the plot was largely symmetrical. However, Egger’s test indicated the presence of publication bias (t = 4.83, p < 0.01). A correction was made using the Trim and Fill Method (TFM) to estimate the true effect size. After correction, the combined effect size remained medium (k = 10, g = 0.79, 95% CI 0.35–1.23, p < 0.01), suggesting that publication bias did not substantially affect the results, and the intervention effect on BMC was close to the true effect size. Sensitivity analysis showed that excluding each study individually resulted in a minimum pooled effect size of g = 0.69 (95% CI 0.34–1.04, k = 9, p < 0.01) when the study by Rittweger and Felsenberg (2009) (Tibia diaphysis) was excluded, and a maximum pooled effect size of g = 0.83 (95% CI 0.50–1.17, k = 22, p < 0.01) when the study by Rittweger et al. (2005) (Radius epiphysis) was excluded. Given that 0.69 and 0.83 are categorized differently based on somewhat arbitrary cutoffs but are numerically close, these values indicate a reasonable level of sensitivity and suggest that the study results are relatively stable, though further validation in future research remains warranted.

**FIGURE 9:**
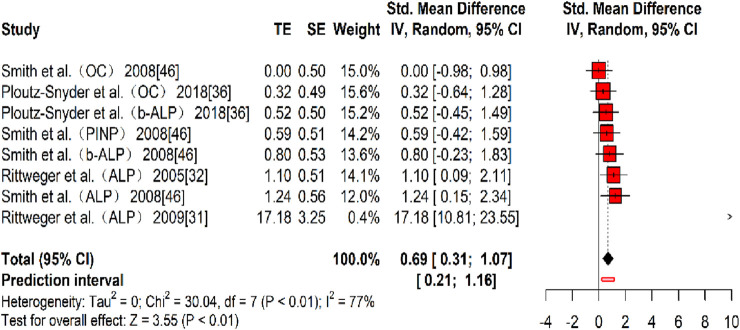
Forest plot of the results for bone formation markers comparing the exercise and control groups before and after resistance training. The values in parentheses indicate the specific bone formation markers analyzed in each study.

Four studies (Rittweger and Felsenberg, 2009; Rittweger et al., 2005; Ploutz-Snyder et al., 2018; Smith et al., 2008) reporting eight effect sizes examined the impact of resistance training on bone formation markers, with a total of 134 participants. The overall Hedges’ g indicated a large effect size (k = 8, g = 0.69, 95% CI 0.31–1.07, p < 0.01) with moderate heterogeneity (τ^2^ = 0, p > 0.01, I^2^ = 77%). The prediction interval ranged from g = 0.21 to 1.16, suggesting that the effect size may vary significantly across different settings (Figure 9). Bias tests were not conducted because the number of effect sizes was fewer than 10 (Sterne et al., 2011).

**FIGURE 10:**
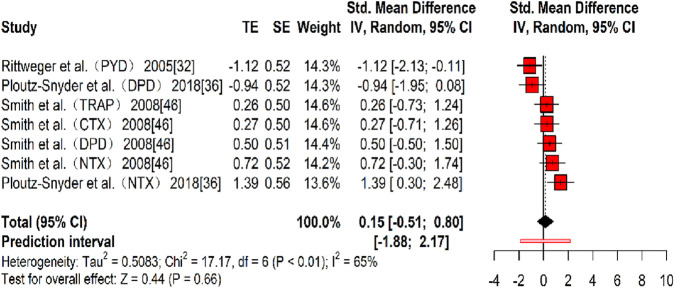
Forest plot of the results for bone resorption markers comparing the exercise and control groups before and after resistance training. The values in parentheses indicate the specific bone resorption markers analyzed in each study.

Three studies (Rittweger et al., 2005; Ploutz-Snyder et al., 2018; Smith et al., 2008) provided seven effect sizes examining the impact of resistance training on bone resorption markers. No significant effects were observed (k = 7, g = 0.15, 95% CI −0.51 to 0.80, p > 0.01) with moderate heterogeneity (τ^2^ = 0.508, p > 0.01, I^2^ = 65%), suggesting no significant difference between the experimental and control groups (Figure 10). Bias tests were not conducted due to the number of effect sizes being fewer than 10 (Sterne et al., 2011).

### 3.5 Moderating variables analysis

Due to the moderate to high heterogeneity observed in the meta-analysis of the preventive effects on muscle atrophy, three dimensions—muscle cross-sectional area (τ^2^ = 1.008, p < 0.01, I^2^ = 70%), muscle volume (τ^2^ = 0.403, p < 0.01, I^2^ = 58%), and muscle strength (τ^2^ = 3.624, p < 0.01, I^2^ = 80%)—were examined with respect to five moderator variables: sex, type of training, training volume, training frequency, and muscle tested. The results of the moderator analyses are presented in Table 3.

**TABLE 3:** Results of a test of moderating variables in resistance training interventions for muscle atrophy.

	Muscle cross-sectional area				Muscle volume				Muscle strength			
Subgroup	Hedges’ *g*	*k*	*n*	*p-value*	Hedges’ *g*	*k*	*n*	*p-value*	Hedges’ *g*	*k*	*n*	*p-value*
*Overall*	0.95(0.50 to 1.39)^a^	27	405	-	0.84(0.57 to 1.12)^a^	36	620	-	2.26(1.42 to 3.11)^a^	23	377	-
Sex												
Male	1.06 (0.43–1.96)^a^	20	290	(*Q* = 1.64; *df* (*Q*) = 2; *p* = 0.44)	0.77 (0.51–1.04)^a^	34	588	(*Q* = 4.19; *df* (*Q*) = 1; *p* = 0.04) **#**	1.17 (1.96–5.02)^a^	8	135	(*Q* = 9.62; *df* (*Q*) = 2; *p* < 0.01) **#**
Female	0.58 (0.07–1.08)^a^	4	64		2.33 (0.86–3.80)^a^	2	32		3.49 (0.80–1.55)^a^	13	208	
Mixed	0.95 (0.36–1.55)^a^	3	51		-	-	-		0.83 (0.12–1.54)^a^	2	34	
Type of training												
FRT	1.93 (0.11–3.75)^a^	5	102	(*Q* = 1.62; *df* (*Q*) = 2; *p* = 0.45)	0.90 (0.61–1.18)^a^	7	516	(*Q* = 9.79; *df* (*Q*) = 2; *p* < 0.01) **#**	1.21 (0.80–1.61)^a^	7	119	(*Q* = 7.11; *df* (*Q*) = 2; *p* = 0.03) **#**
RT	0.73 (0.21–1.25)^a^	15	188		0.13 (-0.44–0.71)	27	72		0.95 (-0.11–2.00)	1	16	
CT	0.73 (0.35–1.12)^a^	7	115		2.33 (0.68–3.80)^a^	2	32		3.08 (1.71–4.45)^a^	15	242	
Training volume												
90days	1.93 (0.11–3.75)^a^	5	102	(*Q* = 2.28; *df* (*Q*) = 2; *p* = 0.32)	0.63 (0.13–1.13)^a^	10	234	(*Q* = 3.77; *df* (*Q*) = 2; *p* = 0.15)	1.21 (0.80–1.61)^a^	7	119	(*Q* = 6.17; *df* (*Q*) = 1; *p* = 0.01) #
60–70days	0.88 (0.52–1.23)^a^	9	147		1.11 (0.77–1.45)^a^	17	280		2.92 (1.63–4.21)^a^	16	258	
20–30days	0.58 (0.03–1.12)^a^	13	156		0.54 (-0.13–1.21)	9	106		-			
Training frequency												
2–3 days/week	1.93 (0.11–3.75)^a^	5	102	(*Q* = 2.28; *df* (*Q*) = 2; *p* = 0.32)	2.33 (0.68–3.80)^a^	2	32	(*Q* = 9.79; *df* (*Q*) = 2; *p* < 0.01) **#**	4.36 (1.20–7.52)^a^	6	96	(*Q* = 2.80; *df* (*Q*) = 1; *p* = 0.09)
3 days/week	0.88 (0.52–1.23)^a^	9	147		0.90 (0.61–1.18)^a^	27	516		1.63 (1.12–2.13)^a^	17	281	
>3 days/week	0.58 (0.03–1.12)^a^	13	156		0.13 (-0.44–0.71)	7	72					
Muscle tested												
Hamstrings&	0.14 (-0.30–0.58)	7	87	(*Q* = 18.16; *df* (*Q*) = 3; *p* < 0.01) **#**	0.16 (-0.10–0.43)	14	240	(*Q* = 41.79; *df* (*Q*) = 3; *p* < 0.01) **#**	1.02 (-0.42–2.45)	2	32	(*Q* = 5.66; *df* (*Q*) = 3; *p* = 0.13)
Quadriceps&	0.42 (-0.14–0.98)	5	74		1.62 (1.19–2.04)^a^	8	126		3.07 (1.55–4.60)^a^	12	197	
Triceps surae&	2.29 (1.40–3.19)^a^	9	138		1.26 (0.87–1.65)^a^	7	133		1.21 (0.22–2.20)^a^	6	99	
Rest of the muscles	0.60 (-0.02–1.22)	6	106		0.93 (0.15–1.71)^a^	7	121		2.86 (0.17–5.56)^a^	3	49	

^a^ Significant difference within a group, # significant difference between groups, &Muscle strength aspects refer to the dominant force-generating muscle groups, FRT, flywheel resistance training; RT, resistance training; CT, concurrent training.

For measures of muscle cross-sectional area, no statistical significance was found in the sex, type of training, training volume, and training frequency subgroups (p > 0.05). However, statistical significance was observed in the muscle tested subgroup (Q = 18.16, df = 3, p < 0.01), with the triceps surae showing a large effect size (k = 9, g = 2.29, 95% CI 1.40–3.19, p < 0.01). In contrast, no significant improvements were found in the hamstrings, quadriceps, or other muscle groups (p > 0.01).

For muscle volume, females (k = 2, g = 2.33, 95% CI 0.86–3.80, p < 0.01) exhibited a greater effect size than males (k = 34, g = 0.77, 95% CI 0.51–1.04, p < 0.01). Within the Type of Training subgroup, concurrent training (CT) (k = 2, g = 2.33, 95% CI 0.68–3.80, p < 0.01) and flywheel resistance training (FRT) (k = 7, g = 0.90, 95% CI 0.61–1.18, p < 0.01) induced large effect sizes, while resistance training (RT) showed no effect (k = 27, g = 0.13, 95% CI −0.44–0.71, p > 0.01). For training frequency, 2–3 days per week (k = 2, g = 2.33, 95% CI 0.68–3.80, p < 0.01) and 3 days per week (k = 27, g = 0.90, 95% CI 0.61–1.18, p < 0.01) resulted in large effect sizes, while >3 days per week showed no effect (k = 7, g = 0.13, 95% CI −0.44–0.71, p > 0.01). Regarding muscle groups, the quadriceps (k = 8, g = 1.62, 95% CI 1.19–2.04, p < 0.01), triceps surae (k = 7, g = 1.26, 95% CI 0.87–1.65, p < 0.01), and other muscles (k = 7, g = 0.93, 95% CI 0.15–1.71, p < 0.01) produced large effect sizes, while the hamstrings showed no effect (k = 14, g = 0.16, 95% CI −0.10–0.43, p > 0.01). However, no statistical significance was found within the training volume subgroup (Q = 3.77, df = 2, p = 0.15).

For muscle strength, females (k = 13, g = 3.49, 95% CI 0.80–1.55, p < 0.01) exhibited a larger effect size than males and mixed groups. Concurrent training (CT) (k = 15, g = 3.08, 95% CI 1.71–4.45, p < 0.01) and flywheel resistance training (FRT) (k = 7, g = 1.21, 95% CI 0.80–1.61, p < 0.01) induced large effect sizes, while resistance training (RT) had no effect (k = 1, g = 0.95, 95% CI −0.11–2.00, p > 0.01). A training duration of 60–70 days (k = 16, g = 2.92, 95% CI 1.63–4.21, p < 0.01) resulted in greater effect sizes compared to 90 days (k = 7, g = 1.21, 95% CI 0.80–1.61, p < 0.01). However, no statistical significance was observed within the training frequency subgroup (Q = 2.80, df = 1, p = 0.09) or the muscle tested subgroup (Q = 5.66, df = 3, p = 0.13).

### 3.6 GRADE level of evidence

As shown in Table 4, the meta-analytic evidence for the effects of resistance training on the prevention of simulated weightlessness is 'high’ for muscle volume and muscle strength, 'moderate’ for muscle cross-sectional area and bone mineralization, and 'low’ for both bone formation and bone resorption markers.

**TABLE 4:** Grade of evidence for resistance training to prevent muscle atrophy and bone loss in simulated weightless populations.

Outcome indicator	Number of effects included	Inconsistent results	Circumstantial evidence	Inaccurate results	Publication bias	Other	Experimental group	Control group	SMD	Level of evidence
muscle cross-sectional area	27	Serious	Not serious	Not serious	Serious	none	181	224	g = 0.95, 95% CI 0.50–1.39, p < 0.01	⊕⊕ΟΟ Moderate
muscle volume	36	Not serious	Not serious	Not serious	Serious	none	276	344	g = 0.84, 95% CI 0.57–1.12, p < 0.01	⊕⊕⊕Ο High
muscle strength	23	Serious	Not serious	Not serious	Serious	none	183	388	2.26, 95% CI 1.42–3.11, p < 0.01	⊕⊕⊕Ο High
Bone Mineral Content	10	Not serious	Not serious	Serious	Serious	none	86	86	g = 0.73, 95% CI 0.41–1.05, p < 0.01	⊕⊕ΟΟ Moderate
bone formation marker	8	Serious	Not serious	Serious	not applicable	none	66	68	g = 0.69, 95% CI 0.31–1.07, p < 0.01	⊕ΟΟΟ Low
Bone resorption markers	7	Serious	Not serious	Serious	not applicable	none	57	59	g = 0.15, 95% CI -0.51–0.80, p > 0.01)	⊕ΟΟΟ Low

## 4 Discussion

### 4.1 Main effects

When astronauts enter the space station, the weightless environment in space can disrupt the homeostasis of the body’s internal systems, leading to multi-system dysfunction, with the locomotor system being the most affected (Lee et al., 2022). The imbalance in neuromuscular control caused by weightlessness weakens the muscle control of movement and reduces muscle strength. This occurs because weightlessness decreases or eliminates the gravitational stimulation of muscle fibers, inhibits the expression of the calcium-binding protein D28K, and reduces the buffering capacity of Ca2+. As a result, Ca2+ overload occurs in muscle fibers, which disrupts sensory nerve endings, weakens muscle nerve conduction, and ultimately inhibits the feedback regulatory pathway between peripheral receptors, the central nervous system, and the muscles, leading to muscle atrophy (Asano et al., 2019). Another key mechanism underlying weightless muscle atrophy is the disruption of the balance between muscle protein synthesis and catabolism. Specifically, abnormalities in the protein synthesis signaling pathway and protease catabolic systems in skeletal muscle cells under weightlessness lead to a decrease in protein synthesis and an increase in protein catabolism, ultimately contributing to muscle atrophy. Muscle atrophy can impair astronauts’ ability to perform daily tasks and may pose a significant risk to their health and safety.

This study found that resistance training increased muscle cross-sectional area (mCSA) under simulated weightlessness, although the effect might have been overestimated. Muscle atrophy or hypertrophy is not uniform along the muscle length, whereas mCSA is typically assessed at a single site, usually near the muscle belly. Consequently, regional variations in adaptation may not be fully captured by mCSA. For example, Miokovic et al. (2012) reported that during 60 days of bed rest, atrophy occurred heterogeneously across different regions: distal quadriceps and biceps femoris (15%–70%), semitendinosus/semimembranosus (∼50%), tibialis anterior (10%–55%), and gastrocnemius (30%–100%). Such non-uniform morphological changes suggest that muscle volume—which accounts for the entire muscle length—may provide a more comprehensive representation of adaptation. Therefore, the significant effect observed for muscle volume but not for mCSA may reflect longitudinal dimensional changes (e.g., muscle length or shape alterations) induced by resistance training under unloading conditions.

In contrast, our study found that resistance training significantly increased muscle volume and muscle strength, with a large effect size in the simulated weightless population. The underlying mechanism may involve resistance training preventing weightless muscle atrophy by improving neuromuscular control, promoting recovery of muscle fiber ultrastructure, and maintaining the balance between protein synthesis and catabolism. First, resistance training induces structural and functional adaptations within muscle spindles, including increased intrafusal fiber size and improved organization of sensory endings (Kröger and Watkins, 2021)., which enhance afferent feedback and reflex sensitivity. These adaptations strengthen the coupling between peripheral proprioceptors and motor neurons, thereby improving neuromuscular control and helping to prevent muscle atrophy under unloading or disuse conditions. For instance, Salles et al. (2015) reported that 8 weeks of strength training significantly enhanced shoulder joint position sense and neuromuscular control in healthy men, supporting the notion that resistance exercise improves proprioceptive function through peripheral and central adaptations.

First, resistance training improves the structure of the muscle spindle and enhances the contractile function of muscle fibers, thereby increasing neural activity between peripheral receptors and muscles to prevent muscle atrophy. For instance, Salles et al. (2015) observed that 8 weeks of strength training significantly increased shoulder joint position sense sensitivity and further improved neuromuscular control in 90 healthy men. Second, resistance training stimulates the synthesis of thick myofilament myosin and thin myofilament actin, which in turn enhances muscle tone. Third, resistance training can promote muscle protein synthesis and inhibit proteolysis, helping to prevent muscle atrophy. Exercise improves ribosome biogenesis by activating mTORC, upregulating phosphorylation levels of its downstream markers (e.g., p70S6KT389 and 4eBP1T36/45), enhancing mRNA translation, and consequently boosting protein synthesis (Ogasawara and Suginohara, 2018). Moreover, resistance training can reduce the expression of important E3 ligases in the ubiquitin-proteasome system (e.g., atrogin1 and MuRF-1), further promoting muscle protein synthesis and preventing muscle atrophy.

Weightlessness-induced bone loss in load-bearing bones is a major health concern for astronauts. The weightless environment leads to osteoblast dysfunction, abnormal bone metabolism, and altered expression of microRNAs, resulting in decreased osteoblast differentiation and increased osteoclast differentiation. This imbalance promotes bone resorption, inhibits bone formation, and disrupts bone homeostasis (Emily and David, 1978). Bone formation markers and bone resorption markers serve as indicators of bone tissue metabolism. Bone formation markers, such as alkaline phosphatase (ALP), bone-specific alkaline phosphatase (b-ALP), osteocalcin (OC), and procollagen type I N-terminal propeptide (P1NP), directly reflect osteoblast function and activity. Elevated levels of these markers suggest enhanced osteoblast activity and an active bone formation process (Zhang and Ma, 2023). For instance, ALP is an enzyme crucial in bone formation, with its activity level correlating to the rate of bone formation. By measuring ALP levels, the rate of bone formation and the growth and repair of bones can be assessed (Zhang et al., 2014). Bone resorption markers, including C-terminal cross-linked peptide (CTX), amino-terminal cross-linked telopeptide of type I collagen (NTX), tartrate-resistant acid phosphatase (TRAP), pyridinoline (PYD), and deoxypyridinoline (DPD), directly indicate bone resorption by osteoclasts. Elevated levels of these markers signal significant bone degradation and decreased bone strength (Zhang and Ma, 2023). For example, significant elevations in CTX and NTX suggest rapid bone loss and an increased fracture risk (Zhang et al., 2014). Thus, the balance between bone formation and bone resorption, through synergistic and antagonistic interactions, regulates bone tissue metabolism. If the rate of increase in bone formation markers surpasses that of bone resorption markers, the outcome favors bone production.

The results of this study demonstrated that resistance training significantly enhanced bone formation markers but had no effect on bone resorption markers in the simulated weightless population. This finding suggests that resistance training positively influences bone production, yielding a rate of bone formation that substantially exceeds its degradation, thus promoting bone mineralization. Furthermore, the study confirmed that resistance training had a significant positive effect on bone mineralization, supporting the notion that resistance training improves bone quality in a microgravity environment. Regarding the underlying mechanisms, it was proposed that, under microgravity, resistance training provides mechanical stress to the bones, activating mechanoreceptors on osteoblasts. This allows osteoblasts to sense changes in stress, thereby promoting their proliferation and differentiation via intracellular signaling pathways (Sun et al., 2019). This process enhances the osteoblasts’ ability to synthesize and secrete bone matrix, which in turn increases the levels of bone formation markers and stimulates bone mineral production. Additionally, the mechanical stimulation from resistance training also regulates osteoblast function, prompting the secretion of various cytokines and growth factors, such as insulin-like growth factor-1 (IGF-1), which further promotes osteoblast activity, increases bone matrix synthesis and mineralization, and contributes to osteogenesis. Moreover, resistance training under microgravity conditions enhances muscle contraction, with the mechanical force generated by muscle contraction exerting influence on the bones. This stimulates calcium uptake and utilization by the bones, increasing the calcium content in bone tissue (Fan et al., 2023). The elevated calcium content supports the deposition of bone minerals, thereby facilitating osteogenesis. Finally, resistance training enhances muscle strength and mass, and the tension generated during muscle contraction is transmitted to the bone through the tendons. This additional mechanical stimulation activates osteoblasts, further promoting bone formation and elevating bone formation markers.

### 4.2 Moderating variables analysis

Previous studies have suggested gender differences in weightless skeletal muscle atrophy, with females exhibiting a tendency to resist weightlessness-induced mitochondrial dysfunction and skeletal muscle fibrosis. However, skeletal muscle atrophy in females appears to occur somewhat earlier than in males (Trappe et al., 2023). In the present study, a moderation analysis was conducted based on sex, revealing that the intervention was more effective in preventing muscle atrophy and strength loss in females than in males.

The analysis suggests that estrogen plays a role in stimulating collagen synthesis, which increases muscle elasticity and toughness. In a microgravity environment, these properties may help maintain the structural integrity of muscles, slowing the rate of muscle atrophy. Furthermore, differences in the muscle fiber composition between men and women may also contribute to these results (Glenmark et al., 1992). In general, women tend to have a higher proportion of slow-twitch muscle fibers (Nuzzo, 2024), which are characterized by better endurance and fatigue resistance, and are capable of sustaining continuous function, thereby maintaining muscle function during strength training in microgravity. In contrast, men typically have a higher proportion of fast-twitch fibers, which are responsible for generating powerful explosive force. However, this explosive force may be less useful in a microgravity environment, and fast-twitch fibers are more prone to atrophy in the absence of gravitational stimulation. It is important to note that the current study predominantly involved male subjects, with fewer studies conducted on female subjects. Therefore, these results should be interpreted with caution and further research is needed to validate the findings.

In terms of intervention type, the present study found that concurrent training was more effective than single strength training. This aligns with a previous meta-analysis by Li et al. (2022), which demonstrated that, when the total volume of strength training was kept equal, concurrent training was more efficient than isolated strength training for improving lower limb strength. The underlying mechanism may be related to the fact that the cardiovascular system is also affected in a microgravity environment (Scott et al., 2022). The inclusion of aerobic training in concurrent training can improve cardiorespiratory function and maintain good blood circulation, which, in turn, supports muscle repair and growth, thereby alleviating muscle atrophy. Another important aspect is that concurrent training can regulate protein metabolism through multiple pathways. Strength training promotes muscle protein synthesis, while aerobic training enhances protein turnover, renewal, and reduces protein breakdown. In a microgravity environment, maintaining a balance between protein synthesis and catabolism is crucial to prevent muscle atrophy. Furthermore, this study found that flywheel resistance training (FRT) was superior to traditional resistance training. Flywheel resistance training is an innovative method that utilizes a rotating flywheel trainer to combine resistance and centrifugal training. This allows for sufficient resistance while controlling the magnitude of both centrifugal and centripetal contraction loads based on training needs, enabling centrifugal overload training (Berg and Tesch, 1994). Compared to traditional resistance training, flywheel training not only ensures adequate resistance but also enables subjects to exert maximum effort. Additionally, FRT avoids biomechanical leverage issues and utilizes bearings with minimal friction, resulting in nearly identical inertia during both centrifugal and centripetal phases. The muscle overload generated by this centrifugal force far exceeds that of traditional resistance training, making flywheel resistance training a more effective intervention.

Intervention period and frequency are critical factors in the implementation of resistance training protocols, and previous studies have shown inconsistencies in these parameters, which can influence the effectiveness of interventions for muscles in a microgravity environment. The present study found that an intervention period of 60–70 days (approximately 8–10 weeks) produced the greatest effect size and provided the most effective protection for muscles, offering a theoretical foundation for the design of resistance training programs in microgravity environments. According to the theory of periodization, strength gains typically progress through three phases: enhancement, maintenance, and detraining. Longer cycle schedules may delay training adaptation, potentially leading to inertia in the subjects, which could hinder optimal results (Zheng and Meng GZ, 2017). For instance, Belavý et al. (2017) conducted a 13-week resistance training program that involved supine leg presses (4 sets of seven repetitions) with 5-min rest intervals, followed by calf raises (4 sets of 14 repetitions with 2-min rest intervals). The intervention did not significantly affect hamstrings, medial thigh, gastrocnemius, or dorsiflexor atrophy. In microgravity, anabolic pathways take time to activate, so short intervention cycles may not allow sufficient muscle adaptation or protein synthesis to increase strength and mass. Regarding frequency, 2–3 sessions per week were more effective than more frequent training, as muscle strength gains generally require at least 48 h of recovery between sessions (Su et al., 2022).

In terms of muscle group sites, the present study found significant differences in the effectiveness of interventions across various muscle groups, with the best results observed for the quadriceps and triceps surae, and poorer results for the hamstrings. The analysis suggests that these differences may be related to the movement patterns involved in resistance training. In existing research literature, during −6° head-down bed rest in a microgravity environment, common resistance training exercises primarily include supine squats (Akima et al., 2001; Belavý et al., 2017; Rittweger et al., 2013), heel raises (Akima et al., 2003; Kouzaki et al., 2007), and stirrups (Alkner and Tesch, 2004a), with the main forces being knee extension, plantarflexion, and hip flexion. The rectus femoris is engaged during both hip flexion and knee extension, while the gastrocnemius is activated during plantarflexion, making these exercises more targeted and effective for these muscle groups. In contrast, despite the hamstrings’ importance for knee stability and activities like getting out of bed (Ono et al., 2011), the lack of exercises specifically targeting knee flexion and foot dorsiflexion led to relatively little activation of the hamstrings, resulting in a poorer intervention outcome for this muscle group. This finding suggests that future resistance training programs during −6° head-down bed rest in a microgravity environment should incorporate a more comprehensive movement pattern based on muscle contraction characteristics to ensure a balanced and effective training regimen.

In summary, while this meta-analysis provides evidence that resistance training effectively mitigates muscle atrophy and bone loss under simulated microgravity, it is important to acknowledge the gravitational differences across space mission phases. Because musculoskeletal unloading and its time course differ between true microgravity and partial gravity, astronauts spend most mission time in microgravity during transit and aboard the ISS, not in lunar or martian gravity. Therefore, findings from ground-based microgravity analogs (e.g., −6° head-down bed rest) are most directly applicable to the prolonged microgravity experienced during transit/ISS operations, while extrapolation to surface activities on the Moon or Mars requires careful qualification.

### 4.3 Strengths and limitations

This study has several limitations. First, research on resistance training in simulated weightlessness has largely focused on disuse myasthenia, with limited data on bone quality, requiring further verification. Second, publication bias may exist due to reliance on published studies, small sample sizes, and selective reporting; this was partially addressed using the cut-and-patch method. Third, although two researchers independently and blindly assessed study quality, using only the PEDro scale may introduce bias from subjective judgment errors.

## 5 Conclusion

Resistance training in simulated weightlessness significantly improved muscle volume and strength (large effect, high-quality evidence) and increased muscle cross-sectional area (moderate-quality evidence). Effects were strongest in women, with concurrent training, 60–70 days, 2–3 sessions/week, targeting quadriceps and triceps surae. For bone, training improved mineral quality (moderate effect) and formation markers (moderate effect, low-quality evidence) but not resorption markers. Given limited and variable bone data, larger, high-quality studies are needed.
