# Supplementary material for: Effects of resistance training on preventing muscle atrophy and bone loss in simulated weightless population: a systematic review and meta-analysis
Source: Front Physiol. 2025 Nov 14;16:1694891. doi: 10.3389/fphys.2025.1694891 (PMC12660080; doi:10.3389/fphys.2025.1694891)
Supplement: Supplementary file 1 [file Supplementaryfile1.docx]

Supplementary Material

TABLE S1. Search strategy

| Database | Full search strategy |
| --- | --- |
| PubMed | (("Muscle Atrophy"[Mesh] OR "Muscular Atrophy"[Mesh] OR “Skeletal muscle atrophy”[Title] OR “Muscle atrophy”[Title] OR “Muscle wasting”[Title] OR “Sarcopenia”[Title] OR “Muscle degeneration”[Title] OR “Loss of muscle mass”[Title] OR “Muscle loss”[Title] OR “Disuse muscle”[Title] OR “Disuse atrophy”[Title]) AND ("Bone Density"[Mesh] OR “Bone loss”[Title] OR “Bone mass loss”[Title] OR “Bone mineral density”[Title] OR “Osteopenia”[Title] OR “Osteoporosis”[Title] OR “Bone weakening”[Title] OR “Skeletal fragility”[Title] OR “Skeletal health”[Title])) AND (("Simulated Weightlessness"[Mesh] OR “Simulated microgravity”[Title] OR “Weightlessness”[Title] OR “Microgravity”[Title] OR “Unloading”[Title] OR “Bed rest”[Title] OR “Prolonged immobilization”[Title] OR “Spaceflight”[Title] OR “Zero gravity”[Title] OR “Space mission”[Title] OR “Head-down tilt”[Title] OR “Space simulation”[Title] OR “Immobilization”[Title])) AND (("Resistance Training"[Mesh] OR "Plyometric Exercise"[Mesh] OR "Weight Lifting"[Mesh] OR “Strength training”[Title] OR “Weight training”[Title] OR “Resistance training”[Title] OR “Resistance exercise”[Title] OR “Concurrent training”[Title] OR “Muscle strengthening”[Title] OR “Hypertrophy”[Title] OR “Exercise program”[Title] OR “Weight-bearing”[Title] OR “Muscular strength”[Title] OR “Isoinertial”[Title] OR “Plyometric”[Title] OR “Stretch shortening exercise*”[Title] OR “Shortening contraction”[Title] OR “Reactive strength”[Title] OR “Power training”[Title] OR “Weightlifting”[Title] OR “Weight lifting”[Title] OR “Explosive strength”[Title] OR “Ballistic”[Title] OR “TRX suspension training”[Title] OR “Suspension training”[Title] OR “Free weight training”[Title] OR “Flywheel”[Title] OR “Eccentric training”[Title] OR “Eccentric contraction”[Title] OR “Eccentric exercis*”[Title] OR “Eccentric-weight”[Title] OR “Eccentric load*”[Title] OR “Negative muscle work”[Title])) AND (("Prevention"[Mesh] OR "Prevention & Control"[Subheading] OR “Prevent muscle atrophy”[Title] OR “Prevent bone loss”[Title] OR “Countermeasure”[Title] OR “Protect muscle”[Title] OR “Protect bone”[Title] OR “Mitigation”[Title] OR “Rehabilitation”[Title] OR “Preservation of muscle”[Title] OR “Preservation of bone”[Title])) |
| Web of Sciences (all databases) | ((TI=(“Muscle atrophy” OR “Muscular atrophy” OR “Skeletal muscle atrophy” OR “Muscle wasting” OR “Sarcopenia” OR “Muscle degeneration” OR “Loss of muscle mass” OR “Muscle loss” OR “Disuse muscle” OR “Disuse atrophy”)) AND TI=(“Bone loss” OR “Bone mass loss” OR “Bone mineral density” OR “Osteopenia” OR “Osteoporosis” OR “Bone weakening” OR “Skeletal fragility” OR “Skeletal health”)) AND TS=(“Simulated microgravity” OR “Weightlessness” OR “Microgravity” OR “Unloading” OR “Bed rest” OR “Prolonged immobilization” OR “Spaceflight” OR “Zero gravity” OR “Space mission” OR “Head-down tilt” OR “Space simulation” OR “Immobilization”) AND TS=(“Resistance training” OR “Strength training” OR “Weight training” OR “Resistance exercise*” OR “Concurrent training” OR “Muscle strengthening” OR “Hypertrophy” OR “Exercise program*” OR “Weight-bearing” OR “Muscular strength” OR “Isoinertial” OR “Plyometric” OR “Stretch shortening exercise*” OR “Shortening contraction” OR “Reactive strength” OR “Power training” OR “Weightlifting” OR “Weight lifting” OR “Explosive strength” OR “Ballistic” OR “TRX suspension training” OR “Suspension training” OR “Free weight training” OR “Flywheel” OR “Eccentric training” OR “Eccentric contraction” OR “Eccentric exercis*” OR “Eccentric-weight” OR “Eccentric load*” OR “Negative muscle work”) AND TS=(“Prevention” OR “Prevent muscle atrophy” OR “Prevent bone loss” OR “Countermeasure” OR “Protect muscle” OR “Protect bone” OR “Mitigation” OR “Rehabilitation” OR “Preservation of muscle” OR “Preservation of bone”) |
| Scopus | TITLE (“Muscle atrophy” OR “Muscular atrophy” OR “Skeletal muscle atrophy” OR “Muscle wasting” OR “Sarcopenia” OR “Muscle degeneration” OR “Loss of muscle mass” OR “Muscle loss” OR “Disuse muscle” OR “Disuse atrophy”) AND TITLE (“Bone loss” OR “Bone mass loss” OR “Bone mineral density” OR “Osteopenia” OR “Osteoporosis” OR “Bone weakening” OR “Skeletal fragility” OR “Skeletal health”) AND ALL (“Simulated microgravity” OR “Weightlessness” OR “Microgravity” OR “Unloading” OR “Bed rest” OR “Prolonged immobilization” OR “Spaceflight” OR “Zero gravity” OR “Space mission” OR “Head-down tilt” OR “Space simulation” OR “Immobilization”) AND ALL (“Resistance training” OR “Strength training” OR “Weight training” OR “Resistance exercise*” OR “Concurrent training” OR “Hypertrophy” OR “Exercise program*” OR “Weight-bearing” OR “Muscular strength” OR “Isoinertial” OR “Plyometric” OR “Stretch shortening exercise*” OR “Shortening contraction” OR “Reactive strength” OR “Power training” OR “Weightlifting” OR “Weight lifting” OR “Explosive strength” OR “Ballistic” OR “TRX suspension training” OR “Suspension training” OR “Free weight training” OR “Strengthening” OR “Flywheel” OR “Eccentric training” OR “Eccentric contraction” OR “Eccentric exercis*” OR “Eccentric-weight” OR “Eccentric load*” OR “Negative muscle work”) AND ALL (“Prevention” OR “Prevent muscle atrophy” OR “Prevent bone loss” OR “Countermeasure” OR “Protect muscle” OR “Protect bone” OR “Mitigation” OR “Rehabilitation” OR “Preservation of muscle” OR “Preservation of bone”) |
| SportDiscus | ( TI (“Muscle atrophy” OR “Muscular atrophy” OR “Skeletal muscle atrophy” OR “Muscle wasting” OR “Sarcopenia” OR “Muscle degeneration” OR “Loss of muscle mass” OR “Muscle loss” OR “Disuse muscle” OR “Disuse atrophy”) ) AND ( TI (“Bone loss” OR “Bone mass loss” OR “Bone mineral density” OR “Osteopenia” OR “Osteoporosis” OR “Bone weakening” OR “Skeletal fragility” OR “Skeletal health”) ) AND ( TX (“Simulated microgravity” OR “Weightlessness” OR “Microgravity” OR “Unloading” OR “Bed rest” OR “Prolonged immobilization” OR “Spaceflight” OR “Zero gravity” OR “Space mission” OR “Head-down tilt” OR “Space simulation” OR “Immobilization”) )  AND ( TX (“Resistance training” OR “Strength training” OR “Weight training” OR “Resistance exercise*” OR “Concurrent training” OR “Hypertrophy” OR “Exercise program*” OR “Weight-bearing” OR “Muscular strength” OR “Isoinertial” OR “Plyometric” OR “Stretch shortening exercise*” OR “Shortening contraction” OR “Reactive strength” OR “Power training” OR “Weightlifting” OR “Weight lifting” OR “Explosive strength” OR “Ballistic” OR “TRX suspension training” OR “Suspension training” OR “Free weight training” OR “Strengthening” OR “Flywheel” OR “Eccentric training” OR “Eccentric contraction” OR “Eccentric exercis*” OR “Eccentric-weight” OR “Eccentric load*” OR “Negative muscle work”) ) AND ( TX (“Prevention” OR “Prevent muscle atrophy” OR “Prevent bone loss” OR “Countermeasure” OR “Protect muscle” OR “Protect bone” OR “Mitigation” OR “Rehabilitation” OR “Preservation of muscle” OR “Preservation of bone”) ) |
